# Review of *The Omnivore’s Deception: What We Get Wrong About Meat, Animals, and Ourselves*

**DOI:** 10.1017/awf.2026.10067

**Published:** 2026-02-12

**Authors:** Cynthia Joanne Naydani

**Affiliations:** The University of Edinburgh Royal Dick School of Veterinary Studies, United Kingdom

Is the human consumption of animal products fundamentally incompatible with living morally and ethically? For John Sanbonmatsu, American philosopher and author of *The Omnivore’s Deception*, the answer is a resounding “yes”. Along the moral spectrum from ‘right’ to ‘wrong’, Sanbonmatsu assigns the use of animals in global food systems firmly and unequivocally into the latter category. As its title suggests, the book is framed largely as a rebuttal to Michael Pollan’s *The Omnivore’s Dilemma* (2006), arguing against Pollan’s proposal that it is possible to both care for, and eat, animals. Sanbonmatsu is not the first to take this stance. From Peter Singer’s foundational *Animal Liberation* (1975) to Jonathan Safran Foer’s *Eating Animals* (2009), many authors have made the moral case for veganism. Sanbonmatsu’s contribution, however, goes beyond the familiar evidence-base that focuses largely on animal rights, extending into an interrogation of the ideological and political structures that allow animal consumption to remain morally palatable.

The book is nicely structured into four parts, each consisting of three chapters. In Part I: Meat in Crisis, Sanbonmatsu interrogates the impact of industrial agriculture on the environment, discussing the climate crisis, loss of biodiversity, and pollution: the trifecta constituting the triple planetary crisis. Capitalism is rightfully identified and explained as the underlying driver of these industrialised approaches, and their subsequent harms. However, Sanbonmatsu disagrees with those, including Pollan, who suggested a return to small-scale agriculture could allow animals to live good lives while ultimately continuing to play a role in human food systems. In Part II: Lies of Dominion, the author links what he considers a growing appeal of pastoralist and agrarian livelihoods with far-right political leanings and slavery, proposing that “*the same arguments once used to legitimate human slavery have been revived and put into play again, this time by apologists for the “new” meat economy”* (p114). This argument is extended in Chapter 6, entitled ‘Love me, Beat me, Kill me, Eat me’ by drawing parallels between livestock farming and domestic violence. In Part III: Lies of Killing, Sanbonmatsu proposes that *“the ‘double helix’ of human supremacism and capitalism… is rooted in masculinist violence, and the same is true of the animal economy through which the two systems find their confluence*” (p140). This section goes on to discuss animal agriculture as a tool for female empowerment. Sanbonmatsu concludes that “*all of this violence suggests nothing so much as a wish on the part of the women to be accepted as co-equals in the death cult of patriarchy*” (p157). Chapter 8, ‘Harry Lime Disease’, integrates psychology, including psychopathy and both inter- and intra-specific empathy, discussing how these can contribute to, or detract from, production and consumption of animal-source foods. The book concludes with Part IV: Beyond Meat, which shifts the conversation away from why we eat animals, to why they should not be eaten. Here, Sanbonmatsu provides a robust case for animal sentience and consciousness. He does this both through provision of the evidence base in a matter akin to what others, such as primatologist Frans de Waal, have done before, interweaving his own lived experiences with his companion animals to provide a rich and multifaceted narrative.

Sanbonmatsu’s book makes for an engaging read that provokes emotion as well as self-reflection. His arguments are thoughtfully constructed and compelling, going as far back as Aristotle’s hierarchical *Scala Naturae*, through to the contemporary rise of “*memoirs by novice women farmers*” (p143). His integration of historical, cultural, geopolitical, and ideological drivers will certainly resonate with those who have already embraced a vegan lifestyle or are contemplating this dietary shift and are looking for a motivational boost.

However, Sanbonmatsu’s holistic, systems-based approach at interrogating the moral harms associated with eating animals contrasts with his comparatively neoliberal framing of dietary habits as a personal choice. The underlying narrative in Sanbonmatsu’s book seems to be that individuals are wholly responsible for deciding what (and who) they eat, presuming that each person has the opportunity to undertake what, for many, would be a radical dietary shift. While reading the book, my impression was that Sanbonmatsu was not addressing *how* people are meant to fully eschew animal products when their own socioeconomic, cultural, geographic, or other circumstances may render this an impossibility. He does briefly acknowledge that “*millions of people in the underdeveloped global South raise and keep animals… because animal husbandry remains one of the few sources of livelihood available to them within a cruel international division of labour*” (p108) but does not offer solutions to this inequity of opportunity. Though this may have been outside the scope of this book, the author’s positivist epistemological stance runs the risk of ostracising those who may feel vilified by this approach.

Sanbonmatsu’s strong positionality extends to criticising the animal welfare scientists who work to improve the lives of the animals who are currently a cornerstone of global food systems. He believes these efforts are misguided and fail to address what he considers the fundamental issue: animals should not, under any circumstances, be a part of the human food chain. By going so far as naming (and criticising) individual animal welfare scientists who have dedicated their lives to bettering those of animals, Sanbonmatsu’s book potentially creates division, rather than collaboration. I would argue that the latter approach is potentially more beneficial, as there are many ways to make a difference, all of which are valid and important.

Overall, *The Omnivore’s Deception* makes for an enlightening and confronting read that could serve as a fantastic catalyst for lively debates and discussions. Sanbonmatsu’s rigorous dissection of the multifaceted factors that drive animal consumption will appeal particularly to those with an interest in animal ethics who are looking for a robust evidence-base. It will likely also encourage readers who are contemplating veganism to take the leap. Yet for animal welfare scientists in particular, the book offers less reassurance than provocation. By casting welfare science as a moral compromise rather than a progressive force, Sanbonmatsu considers this discipline to be part of the problem. Whether or not readers accept this critique, *The Omnivore’s Deception* succeeds in prompting contemplation regarding the ethical limits of reformist approaches to animal use.
